# A “Pandemic-Proof” Methodology for Outbreak Detection Adapted From COVID-19’s Impact on Notifications of Infectious Diseases in the Netherlands: Surveillance Study

**DOI:** 10.2196/73953

**Published:** 2025-08-26

**Authors:** Babette van Deursen, Stijn Raven, Wolfer van den Bosch, Cornelia H M van Jaarsveld, Aura Timen

**Affiliations:** 1 Department of Primary and Community Care, Research Institute for Medical Innovation, Radboud University Medical Center, Geert Grooteplein 21, Nijmegen, 6500 HB, The Netherlands, +31622989091; 2 Department of Infectious Disease Control, Public Health Service region Utrecht, Zeist, The Netherlands

**Keywords:** public health surveillance, COVID-19 pandemic, infectious diseases, epidemiology, outbreak detection, routine surveillance data

## Abstract

**Background:**

Reporting of notifiable infectious diseases was overall impacted by the COVID-19 pandemic. This could affect disease surveillance and thus, outbreak detection, as it is based on historical data. For effective outbreak detection, it is crucial that communicable disease control professionals can rely on accurate and valuable alarm thresholds to ensure timely and adequate response to potential outbreaks.

**Objective:**

In this study, we take the first steps in the development of a methodology that adjusts for the impact of the COVID-19 pandemic on the number of notifications of notifiable infectious diseases and provides corrected alarm thresholds for outbreak detection. We identify which infectious diseases were affected by the COVID-19 pandemic, assess the duration of these effects, and explore potential correction methods to improve outbreak detection.

**Methods:**

We analyzed cases of 25 notifiable infectious diseases reported from 2015‐2023 in the Netherlands. Negative binomial regression was used to calculate the incidence rate ratios for each period: pre-COVID-19 pandemic, COVID 2020, COVID 2021, COVID 2022, and post-COVID-19 pandemic. To address the decrease in notifications during the COVID-19 pandemic, we tested 3 correction methods: (1) recoding COVID-19 years as missing; (2) imputing with the last pre-COVID-19 pandemic observation; and (3) imputing the historical moving average.

**Results:**

Between 2015‐2023, a total of 74,990 notifications were reported in the Netherlands, of which 9,836 notifications (13%) occurred during the COVID-19 pandemic. Malaria, typhoid fever, hepatitis A, meningococcal infection, paratyphoid fever, Q-fever, shigellosis, measles, mumps, and pertussis had significantly lower notifications during the COVID-19 pandemic, but the duration and magnitude of the effect differed among the infections. The effect of the COVID-19 pandemic on the notifications of malaria (incidence rate ratio [IRR] 0.17, 95% CI 0.08‐0.36) and typhoid fever (IRR 0.12, 95% CI 0.02‐0.55) was only seen in 2020, while the notifications for measles (IRR 0.34, 95% CI 0.15‐0.75) and pertussis (IRR 0.34, 95% CI 0.20‐0.60) were still significantly lower in the post-COVID-19 pandemic period. In addition, the newly calculated alarm thresholds showed a noticeable difference compared with the original unadjusted alarm thresholds. However, the variation among the 3 different corrected alarm thresholds was not substantial.

**Conclusions:**

During the COVID-19 pandemic, notifications of 10 infectious diseases declined significantly. The duration of this decline varied among infections, highlighting the need for pattern-specific adjustments. Our study demonstrates that accounting for the reduced notifications impacts alarm threshold calculations for outbreak detection. We therefore recommend amending the alarm thresholds to account for this impact to ensure reliable outbreak detection, so that communicable disease control professionals can act in a timely and data-driven manner. The next step in the development of the “pandemic-proof” methodology is to determine which correction method is most suitable. Further validation by communicable disease control professionals is essential to assess the applicability of the adjusted alarm thresholds for outbreak detection.

## Introduction

In the Netherlands, it is mandatory under the Dutch Public Health Act for all medical doctors and laboratories to notify certain infectious diseases to the public health services (PHS), who then report it to the National Institute for Public Health and the Environment (RIVM) [1]. Among others, the RIVM is responsible for the surveillance of those notifiable infectious diseases at the national level and the PHS at a regional level. A critical aspect of surveillance is outbreak detection, for which various methods establish an alarm threshold for the number of notifications that signal for a potential outbreak detection [2-6]. Regarding these methods, robust historical data are essential for optimizing alarm thresholds accuracy. This allows communicable disease control professionals to implement preventive actions or control measures in a timely manner, and at the same time minimizing false positive signals, to effectively interrupt transmission and thus contain an outbreak.

The COVID-19 pandemic had an impact on the notifications of other infectious diseases. Several countries worldwide reported less notifications than expected during the pandemic on all types of infectious diseases, not only respiratory infections [7-16]. Nonpharmaceutical interventions (NPIs) that were implemented to decrease the transmission of SARS-CoV-2 most likely influenced the transmission of other infectious diseases, especially respiratory infections. Some studies suggest that SARS-CoV-2 was in competition with other respiratory infections, which suppressed the transmission of the lesser dominant respiratory infection [17-19]. In addition, the pandemic disrupted health care systems and altered health-seeking behavior, resulting in underdiagnosing and underreporting and thus, a decrease in notifications of other infectious diseases [20].

Since outbreak detection thresholds are based on historical data and considering reports of the impact of the COVID-19 pandemic on notifications of other infectious diseases globally, it is essential to investigate what the effect of the COVID-19 pandemic was on the number of notifications. This includes investigating which infectious diseases were affected and the duration of these effects, and whether these trends align with the aforementioned hypothesized causes. This is crucial for understanding the impact of the COVID-19 pandemic on outbreak detection, as it still remains unknown. To our knowledge, no previous studies have been conducted on how to adjust for the impact of the COVID-19 pandemic on the historical data and thus, establish accurate alarm thresholds for outbreak detection. The development of a methodology that adjusts for the impact of the COVID-19 pandemic on the number of notifications of notifiable infectious diseases requires a stepwise approach: first identifying which infectious diseases were affected by the COVID-19 pandemic, then developing a novel methodology, and finally validating the novel methodology for its applicability in effective outbreak detection. This future “pandemic-proof” methodology will provide corrected historical averages and alarm threshold as indicators of changes in infectious disease trends, to monitor and signal for upcoming infectious disease outbreaks.

In this study, we focus on the first steps of this process toward a “pandemic-proof” methodology by examining which infectious diseases were affected by the COVID-19 pandemic, the duration of these effects, and exploring approaches to correct for them in outbreak detection. The validation of any correction method is beyond the scope of this study, as it first requires a comprehensive understanding of the impact of the COVID-19 pandemic on infectious disease notifications, as well as of the potential correction methods for addressing this.

## Methods

### Study Design and Data Sources

To determine which notifiable infectious diseases were affected by the COVID-19 pandemic on the number of notifications in the Netherlands, we used anonymized routinely collected data on all 45 notifiable infectious diseases from the database “Osiris” collected by National Institute of Public Health and Environment. Notifiable clusters of infections, such as Methicillin-resistant Staphylococcus aureus clusters, were left out of the dataset. All notifications (except COVID-19 pandemic) between January 2015 and December 2023 based on the date of disease onset were included. In addition, information on age, sex, region (the regional distribution is the PHS catchment area), and type of infection for each notification was extracted from the database. Infectious diseases with lacking historical data (eg, notifiable since 2019 or later on) were excluded from the analysis (n=4). Furthermore, we also excluded infectious diseases that were only notified 20 times or less during the entire study period (n=16). We investigated the notifications of 25 infectious diseases.

### Definition of Study Periods

To investigate the duration of the COVID-19 pandemic effect, we split our study period into different periods: pre-COVID-19 pandemic, COVID 2020, COVID 2021, COVID 2022, and post-COVID-19 pandemic. Pre-COVID-19 pandemic was defined as January 2015-February 2020, as the first case in the Netherlands was reported at the end of February 2020 and the first national NPIs were implemented mid-March. COVID 2020 was defined as March 2020-December 2020, COVID 2021 included the whole year of 2021, COVID 2022 was defined as January 2022-August 2022 and post-COVID-19 pandemic was defined as September 2022-December 2023. We chose September 2022 as the ending date of the COVID-19 pandemic, as the COVID-19 stringency index was at its lowest for the Netherlands and all NPIs were not in place anymore [21]. Since then, there were no NPIs implemented in the Netherlands.

### Hypothesis Development

First, we hypothesized what the potential impact of the COVID-19 pandemic was on the number of notifications and trends for every notifiable infectious disease. We based our hypotheses on 4 factors: effect of NPIs (eg, physical distancing and travel bans), variants of SARS-CoV-2 interference (competing between infections), demographic determinants (ie, region, age, and gender), health care use and access to diagnostics. All factors were reviewed by the team with extensive knowledge on infectious disease control (BvD, infectious disease epidemiologist; SR, medical doctor in communicable disease control; CHMvJ, epidemiologist; and AT, professor and medical doctor in communicable disease control) and discrepancies were discussed until consensus was reached. The latter potential factor (access to diagnostics) was additionally reviewed by a microbiologist. The effect of the NPIs was leading in this decision-making, whether we expected an effect on the notifications or not (Multimedia Appendix 1).

### Statistical Analysis

For the descriptive analysis, we calculated the age, sex, and region distribution for each infection in each study period (pre-COVID-19 pandemic, COVID 2020, COVID 2021, COVID 2022, and post-COVID-19 pandemic). To examine which infectious diseases were affected and the duration of these effects, we performed negative binomial regression to determine the incidence rate ratios (IRR) and their respective 95% CI, using pre-COVID-19 pandemic as reference period. The included months of the reference period corresponded to the respective months of the investigated study period to account for seasonality. For example, the COVID 2020 period was compared with the historical months March until December from the years 2015‐2019. We defined the impact of the COVID-19 pandemic on infectious disease notifications as a significantly lower IRR than the pre-COVID period, with the effect beginning in the COVID 2020 period, as we considered that substantial effects of the pandemic should be visible in that period. Effects that emerged later are more difficult to directly attribute to the COVID-19 pandemic.

### Threshold Recalibration

For the exploration of recalibrating the alarm threshold, we reviewed literature and expert opinions on the correction for the decrease in notifications. The correction was integrated into an alarm threshold for outbreak detection, which is mostly based on the method of Stroup that focuses on the moving average [3]. The moving average for a month is calculated by the means of the preceding, current, and upcoming month of the last 5 years, with a corresponding moving SD and moving SE. For example, the moving average of July 2021 is calculated by the means of the months June, July, and August of the years 2016‐2020. If there was an outbreak in one of those months (defined as: moving average+[2*SD]), the number of cases was set back to the moving average of that month. Thus, we calculated the alarm threshold with the moving average that is corrected for outbreaks, from now on called baseline. The original alarm threshold itself is calculated as: baseline+(1.96 * SQRT[SD^2^+SE^2^]).

After assessing which notifiable infectious diseases were affected, we tested the following 3 methods for correction: (1) treating disease counts as missing during the correction period; (2) replacing disease counts with the last value (outbreak corrected) for the same month in the year preceding the correction period; and (3) replacing disease counts with the baseline value (as mentioned above) for the same month in the year preceding the correction period. We calculated new thresholds for infectious diseases impacted by the COVID-19 pandemic, focusing on those with a decrease in notifications, and only for the corresponding affected period. We used the original alarm threshold method (as described above), as a reference. We refer to this further on as the unadjusted threshold method, as it is not corrected for a decreased number of notifications.

### Ethical Considerations

We used anonymized notification data collected by the RIVM in accordance with the Dutch Public Health Act. The Medical Ethics Committee of Radboudumc declared that an ethical approval was not required for this study, as the study did not fall under the scope of the Medical Research Involving Human Subjects Act.

## Results

### Overview

In the entire study period (2015‐2023), 74,990 notifications were reported of the 25 included infectious diseases. Of those 74,990 notifications, 73% (n=54,430) were notified in the pre-COVID-19 pandemic period, 13% (n=9836) during the COVID-19 pandemic, and 14% (n=10,724) in the post-COVID-19 pandemic period. Notification numbers and characteristics for each notifiable infectious disease are provided in (Multimedia Appendix 2). We found no difference regarding age, sex, or region distribution between the periods.

### Affected by the COVID-19 Pandemic

We hypothesized that the notifications of Creutzfeldt-Jakob disease, Q-fever, hantavirus, listeriosis, and psittacosis were not affected by the COVID-19 pandemic (Multimedia Appendix 1). For all other 20 notifiable infectious diseases, we expected a decrease in notifications due to implementation of the NPIs (egsimilar transmission routes), interference of other infections, changing demographics, change in health care seeking behavior or use of diagnostics.

**Figure 1.**
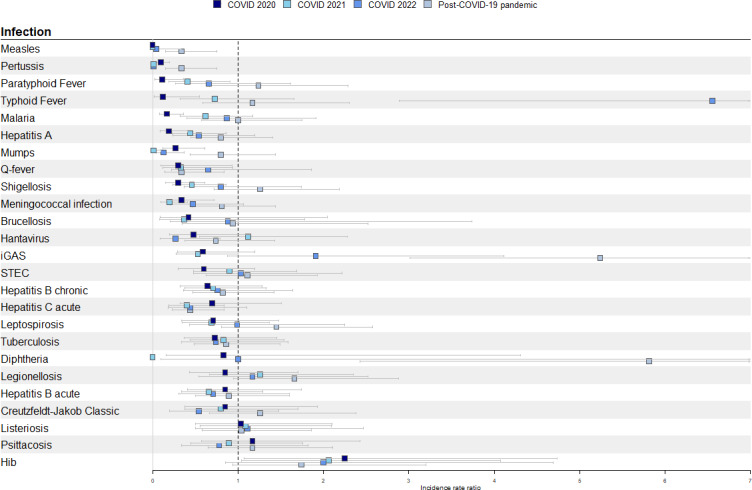
Incidence rate ratios for 25 notifiable infectious diseases plotted by the COVID-19 pandemic periods (2020, 2021, 2022 and post-COVID-19 pandemic), with the pre-COVID-19 pandemic period (January 2015-February 2020) as reference, the Netherlands. Hib: *Haemophilus influenzae* type b; iGAS: invasive group A streptococcal; STEC: Shiga toxin-producing *E. coli*.

Based on the IRRs, we found that the number of notifications of 11 infectious diseases was significantly affected by the COVID-19 pandemic (Table 1 and Figure 1). However, the duration of affected months differed. In Figure 1, we present the IRRs per COVID-19 period for all infectious diseases, indicating whether a significant effect was found and how this effect evolved over time.

**Table 1.** Incidence rate ratios (IRRs) for notifications in the COVID-19 pandemic and post-COVID-19 pandemic periods compared with pre-COVID-19 pandemic for 25 notifiable infectious diseases in the Netherlands, January 2015-December 2023.

Infectious disease	Pre-COVID-19 pandemic (January 2015–February 2020)		COVID 2020 (March-December)		COVID 2021 (January-December)		COVID 2022 (January-August)		Post-COVID-19 pandemic (September 2022–December 2023)	
	Notifications, n	IRR (95% CI)	Notifications, n	IRR (95% CI)	Notifications, n	IRR (95% CI)	Notifications, n	IRR (95% CI)	Notifications, n	IRR (95% CI)
Effect during COVID 2020										
* *Typhoid fever	106	Ref.^a^	2	0.12 (0.02‐0.55)	15	0.73 (0.32‐1.66)	93	6.55 (2.89‐14.86)	32	1.17 (0.59‐2.30)
* *Malaria	1281	Ref.	37	0.17 (0.08‐0.36)	154	0.62 (0.32‐1.17)	138	0.87 (0.40‐1.91)	330	1.00 (0.57‐1.75)
Effect during COVID 2020 and 2021										
Hepatitis A	900	Ref.	29	0.19 (0.09‐0.41)	78	0.44 (0.23‐0.86)	60	0.54 (0.24‐1.20)	185	0.80 (0.45‐1.41)
Meningococcal infection	837	Ref.	44	0.34 (0.16‐0.72)	33	0.20 (0.10‐0.41)	51	0.47 (0.21‐1.06)	175	0.81 (0.46‐1.44)
Paratyphoid fever	210	Ref.	4	0.11 (0.03‐0.36)	17	0.41 (0.19‐0.91)	19	0.66 (0.27‐1.62)	67	1.24 (0.67‐2.29)
Q-fever	92	Ref.	5	0.30 (0.10‐0.93)	6	0.33 (0.12‐0.94)	8	0.65 (0.22‐1.86)	8	0.34 (0.14‐0.84)
Shigellosis	2483	Ref.	129	0.30 (0.15‐0.61)	221	0.46 (0.24‐0.86)	243	0.80 (0.37‐1.74)	807	1.26 (0.72‐2.19)
Hib^b^	174	Ref.	58	2.25 (1.07‐4.74)	67	2.06 (1.04‐4.07)	36	2.00 (0.85‐4.69)	78	1.74 (0.94‐3.20)
Effect during COVID 2020 – 2022										
Mumps	452	Ref.	20	0.27 (0.12‐0.61)	1	0.01 (0.00‐0.10)	7	0.13 (0.04‐0.37)	93	0.80 (0.44‐1.44)
Pertussis	29,037	Ref.	485	0.10 (0.05‐0.20)	79	0.01 (0.00‐0.03)	56	0.01 (0.00‐0.03)	2570	0.34 (0.20‐0.60)
Measles	139	Ref.	0	<0.001 (0.00‐0.00)	0	<0.001 (0.00‐0.00)	1	0.04 (0.01‐0.34)	12	0.34 (0.15‐0.75)
No effect										
Legionellosis	2722	Ref.	413	0.85 (0.43‐1.70)	670	1.26 (0.67‐2.35)	405	1.17 (0.54‐2.52)	1162	1.66 (0.95‐2.88)
Brucellosis	29	Ref.	2	0.42 (0.09‐2.05)	2	0.37 (0.08‐1.78)	3	0.88 (0.21‐3.74)	7	0.94 (0.35‐2.52)
Diphtheria	14	Ref.	2	0.83 (0.16‐4.31)	0	<0.001 (0.00‐0.00)	1	1.00 (0.10‐9.75)	21	5.81 (2.43‐13.89)
Hepatitis B acute	561	Ref.	78	0.85 (0.41‐1.74)	72	0.66 (0.34‐1.29)	50	0.71 (0.31‐1.60)	129	0.89 (0.50‐1.60)
Hepatitis B chronic	5356	Ref.	553	0.64 (0.32‐1.28)	741	0.71 (0.38‐1.33)	519	0.76 (0.36‐1.64)	1129	0.82 (0.47‐1.42)
Leptospirosis	423	Ref.	57	0.71 (0.34‐1.48)	58	0.69 (0.35‐1.37)	51	0.99 (0.43‐2.25)	158	1.45 (0.81‐2.58)
Listeriosis	482	Ref.	83	1.03 (0.50‐2.10)	102	1.09 (0.56‐2.09)	69	1.11 (0.50‐2.47)	129	1.04 (0.58‐1.86)
Psittacosis	342	Ref.	67	1.17 (0.57‐2.43)	56	0.89 (0.45‐1.76)	33	0.78 (0.34‐1.82)	103	1.17 (0.65‐2.11)
STEC^c^	2707	Ref.	286	0.60 (0.30‐1.20)	481	0.90 (0.48‐1.69)	387	1.03 (0.48‐2.22)	772	1.11 (0.63‐1.93)
Tuberculosis	4189	Ref.	506	0.73 (0.37‐1.45)	673	0.83 (0.44‐1.54)	423	0.74 (0.34‐1.59)	925	0.86 (0.49‐1.49)
Creutzfeldt-Jakob classic	154	Ref.	22	0.85 (0.38‐1.93)	24	0.80 (0.38‐1.70)	10	0.54 (0.20‐1.48)	50	1.26 (0.67‐2.38)
Hantavirus	179	Ref.	15	0.48 (0.20‐1.14)	39	1.12 (0.55‐2.28)	7	0.27 (0.09‐0.79)	34	0.74 (0.38‐1.43)
Hepatitis C acute	293	Ref.	33	0.70 (0.32‐1.51)	23	0.40 (0.19‐0.84)	17	0.44 (0.18‐1.10)	33	0.44 (0.23‐0.84)
iGAS^d^	1268	Ref.	110	0.59 (0.29‐1.20)	127	0.53 (0.28‐1.00)	358	1.91 (0.88‐4.11)	1715	5.24 (3.01‐9.12)

a Ref.: Reference.

b Hib: *Haemophilus influenzae* type b.

c STEC: Shiga toxin-producing *E. coli*.

d iGAS: invasive group A streptococcal.

**Figure 2.**
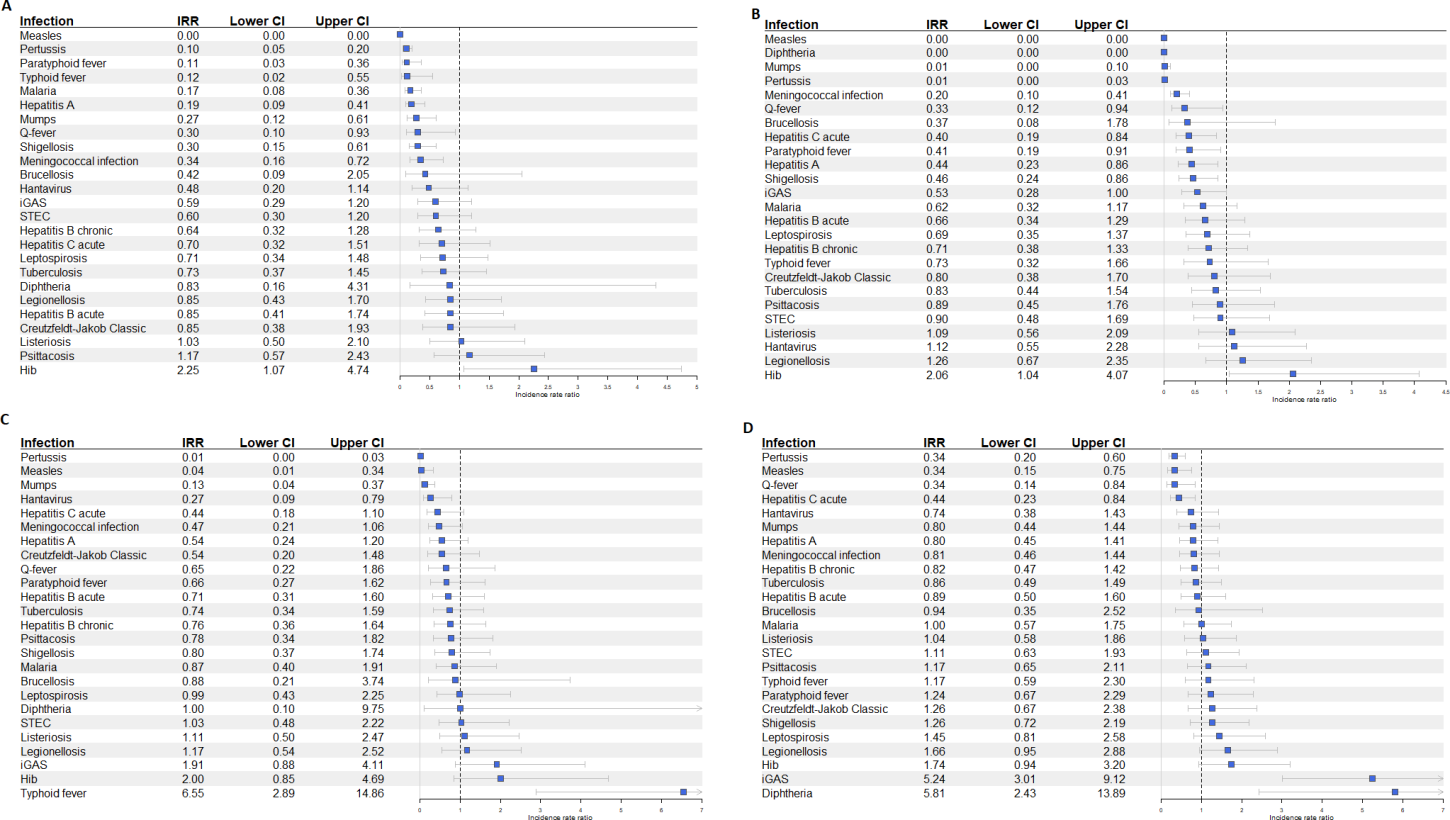
IRRs for 25 notifiable infectious diseases for each COVID-19 pandemic period compared with pre-COVID-19 pandemic period (January 2015-February 2020): (A) COVID 2020 period; (B) COVID 2021 period; (C) COVID 2022 period; and (D) Post-COVID-19 pandemic period, the Netherlands. Hib: *Haemophilus influenzae* type b; iGAS: invasive group A streptococcal; IRR: incidence rate ratio; STEC: Shiga toxin-producing *E. coli*.

Malaria (IRR 0.17, 95% CI 0.08‐0.36) and typhoid fever (IRR 0.12, 95% CI 0.02‐0.55) were significantly less notified during COVID 2020 and were back on pre-COVID-19 pandemic levels in 2021 (Figure 2). Typhoid fever was in the COVID 2022 period significantly more reported compared with the pre-COVID-19 pandemic period, which was related to a large outbreak (IRR 6.55, 95% CI 2.89‐14.86) [22]. Infectious disease notifications affected in 2020 and 2021 were hepatitis A, meningococcal infection, paratyphoid fever, Q-fever, and shigellosis. *Haemophilus influenzae* type b (Hib) was also affected in 2020 and 2021; however, contradictory to other infections, the number of notifications increased significantly in those periods (IRR 2.25, 95% CI 1.07‐4.74 and IRR 2.06, 95% CI 1.04‐4.07, respectively). This significant increase was not reported in the COVID 2022 period (Figure 2).

Mumps, measles, and pertussis were affected during the entire duration of the COVID-19 pandemic, with significantly less notifications reported (Table 1). Furthermore, the notifications for measles and pertussis continued to be low in the post-COVID-19 pandemic period as well (IRR 0.34, 95% CI 0.15‐0.75 and IRR 0.34, 95% CI 0.20‐0.60, respectively). Our hypotheses on whether we expected an effect of the COVID-19 pandemic on the notifications did not match with the found effects for Q-fever and Hib.

Our hypotheses on whether we expected an effect of the COVID-19 pandemic on the notifications did not match with the found effects for Q-fever and Hib.

### Not Affected by the COVID-19 Pandemic

Based on our definition of the impact of the COVID-19 pandemic, we found no effect on the notifications of the other 14 infectious diseases: legionellosis, brucellosis, diphtheria, hepatitis B acute, hepatitis B chronic, leptospirosis, listeriosis, psittacosis, Shiga toxin-producing *E. coli*, tuberculosis, Creutzfeldt-Jakob disease, hantavirus, hepatitis C acute, and invasive Group A streptococcal disease. However, we expected an effect in ten of them (Multimedia Appendix 1). Our hypotheses for hantavirus, listeriosis, psittacosis, and Creutzfeldt-Jakob disease were correct.

### Correcting for the Impact of COVID-19 Pandemic

As the notifications of malaria, typhoid fever, hepatitis A, meningococcal infection, paratyphoid fever, Q-fever, shigellosis, mumps, measles, and pertussis were significantly less during the COVID-19 pandemic, we calculated their adjusted alarm thresholds for each correction method. The alarm thresholds for outbreak detection do not require adjustment for Hib, as Hib notifications increased during the COVID-19 pandemic, and correcting for outbreaks is standard practice in the original method.

**Figure 3.**
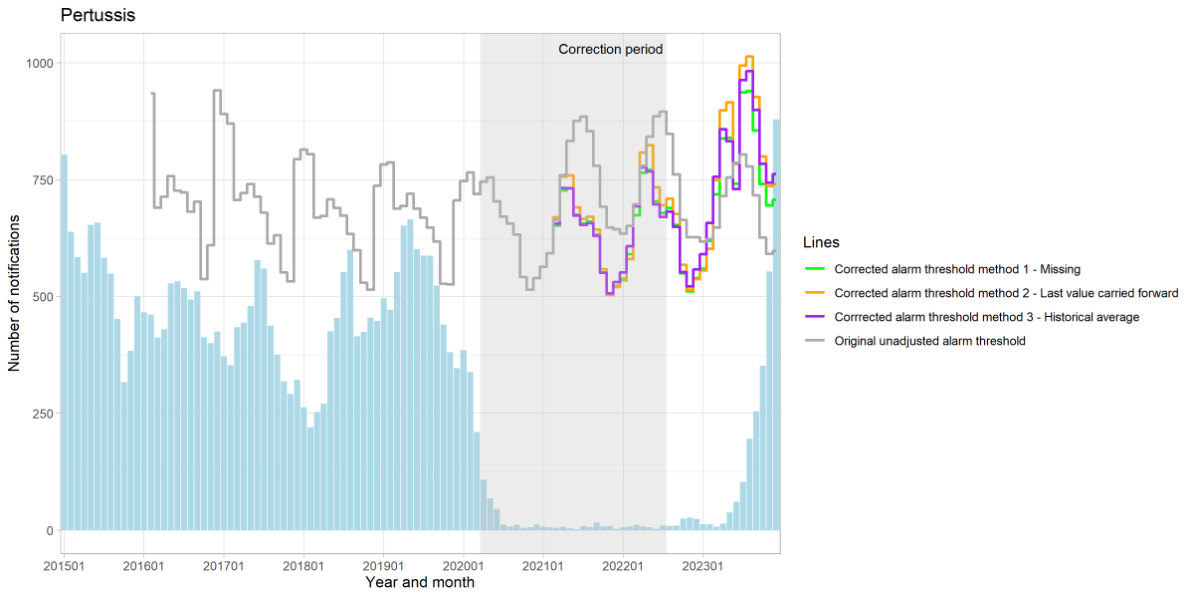
Number of monthly notifications of pertussis with the unadjusted threshold and 3 corrected threshold methods for outbreak detection, the Netherlands, January 2015-December 2023.

The results of the different correction methods are presented in (Figures3  4, Multimedia Appendices 3-10). We found that there is a difference in the number of notifications between the unadjusted and corrected alarm thresholds for exceeding the alarm threshold; however, no clear difference was observed between the different corrected alarm thresholds. The difference in the number of exceedances of the alarm threshold per method compared with the original unadjusted method was only seen for malaria and mumps (Multimedia Appendix 11). We observed that the corrected alarm thresholds are influenced by the duration and magnitude of the effect of the COVID-19 pandemic, as well as the number of notifications. Over the observed study period, we highlight 2 noteworthy results.

**Figure 4.**
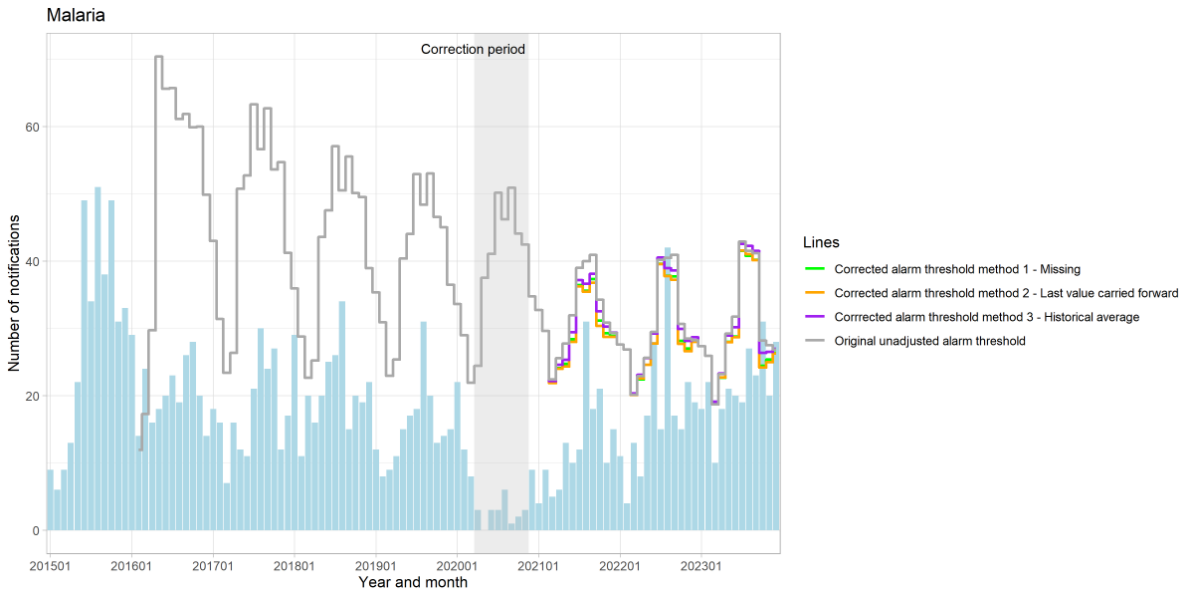
Number of monthly notifications of malaria with the unadjusted threshold and 3 corrected threshold methods for outbreak detection, the Netherlands, January 2015-December 2023.

First, we found that the corrected alarm thresholds for hepatitis A, measles, mumps, paratyphoid fever, and Q-fever were generally higher than their original unadjusted alarm thresholds. The alarm thresholds for typhoid fever showed minimal variation across methods. In contrast, the corrected alarm thresholds were at some points in time lower than the unadjusted alarm thresholds for pertussis, meningococcal infection, and shigellosis. For instance, during the COVID-19 pandemic, the corrected alarm thresholds for pertussis were lower, with a difference of 200 notifications compared with the unadjusted alarm threshold (Figure 3 and Multimedia Appendix 12). This indicates that the correction already impacts the alarm thresholds during the COVID-19 pandemic.

Second, the length of the correction period also varies depending on the duration of the effects of the COVID-19 pandemic on specific disease notifications, which in turn influences the corrected alarm thresholds. Malaria, for example, was only corrected for the period March-December 2020 during which notifications significantly decreased (Figure 4 and Multimedia Appendix 13). As the correction period is longer, the immediate effect of the correction seems more visible compared with a shorter correction period, such as seen with pertussis (Figure 3 and Multimedia Appendix 12). Furthermore, the disparity between the unadjusted and corrected thresholds seems to gradually diminish earlier over time, compared with the infectious diseases with a longer correction period.

## Discussion

### Principal Findings

This study shows that the COVID-19 pandemic had a significant impact on the notifications of other infectious diseases, and therefore on the surveillance and especially a noteworthy effect on the alarm thresholds for outbreak detection. To take timely preventive actions and implement control measures to contain outbreaks, an accurate alarm threshold for outbreak detection is essential. Malaria, typhoid fever, hepatitis A, meningococcal infection, paratyphoid fever, Q-fever, shigellosis, measles, mumps, and pertussis had significantly lower notifications during the COVID-19 pandemic, but the duration of the effect differed among the infections. Only measles, mumps, and pertussis were affected during the whole duration of the COVID-19 pandemic, and for measles and pertussis, this effect continued in the post-COVID-19 pandemic period. Interestingly, the notifications of Hib increased significantly during the COVID-19 pandemic. In addition, the newly calculated alarm thresholds for infectious diseases with decreased notifications showed a noticeable difference compared with the unadjusted alarm thresholds. However, the variation among the 3 different corrected alarm thresholds was not substantial.

### Comparison With Previous Work

Decrease in notifications of malaria, typhoid fever, hepatitis A, meningococcal infection, paratyphoid fever, shigellosis, measles, mumps, and pertussis was expected based on the hypotheses. This was already observed in other countries for malaria, hepatitis A, meningococcal infection, shigellosis, measles, mumps, and pertussis [7-13,15]. In our study, the notifications of Q-fever unexpectedly decreased, while this concerns a zoonotic disease, and the presence of *Coxiella burnetii* in the reservoir in the Netherlands was unlikely to be affected by the COVID-19 pandemic and the NPIs. Furthermore, the number of notifications of the other 14 investigated infectious diseases showed no impact from the COVID-19 pandemic. However, other studies reported a decrease in notification numbers of brucellosis, hepatitis B (acute and chronic), hepatitis C, legionellosis, leptospirosis, listeriosis, and tuberculosis, possibly due to differences in surveillance systems and case definitions [7,8,10,11,13].

Surprisingly, we found a significant increase in the Hib notifications during COVID 2020 and 2021. We expected that Hib would decrease, as it has a similar transmission route as SARS-CoV-2, and therefore, the NPIs could have impacted Hib transmission as well. Besides, other studies described a decrease in Hib notifications during the COVID-19 pandemic [11,23,24]. According to the study of Steens et al [25], the observed increase in incidence in the Netherlands could be explained by methodological, behavioral, biological, or vaccine-related factors; however, the conclusive reason still remains unknown.

In exploring the post-COVID-19 period, we found that the number of notifications for measles and pertussis remained significantly lower compared with the pre-COVID-19 period. This prolonged effect on the notifications was also observed in Poland [26]. This is most likely due to the prolonged unintended effects of NPIs, leading to reduced circulation of these infectious diseases [27]. It highlights the importance of a robust surveillance system, as a resurgence of cases is likely to occur in the future. In the Netherlands, we have experienced a pertussis outbreak in early 2024, exceeding pre-COVID-19 levels (data not shown).

Our study shows that correcting for the impact of the COVID-19 pandemic on the decreased notifications of other notifiable infectious diseases has an effect on the alarm threshold for outbreak detection. The levels of the original unadjusted alarm thresholds differed from the other alarm threshold methods, which were corrected for the decrease in notifications. Other studies that observed a significant decrease in their notifications briefly mention the impact for surveillance; however, none of them propose a concrete solution [11,28]. To our knowledge, this is the first study that investigates whether correction for the decrease in notifications has an effect on outbreak detection and investigates multiple correction methods. Based on our findings, the initial difference among the investigated alarm threshold methods was not evident. This could be explained by the delayed effect of the correction, as it calculates with historical values and could appear visibly later on in the period of 2025‐2027. Nevertheless, the effect was already strongly visible for the infectious diseases with high notification numbers and a longer correction period, such as pertussis. This underlines the need for pattern-specific corrections. However, implementing a different outbreak detection method for every infectious disease would be too complex in a PHS setting and would require more data points than are available, especially for infectious diseases with low notification numbers. We therefore propose a general methodology that incorporates pattern-specific corrections based on the timing of impact. For example, the alarm threshold for malaria would only be corrected for the COVID 2020 period, while the alarm threshold for pertussis will be corrected for the whole COVID-19 pandemic period. The next step will be the validation by communicable disease control professionals to assess the applicability of the corrected alarm thresholds for outbreak detection.

The significant and prolonged effect of the COVID-19 pandemic on notifiable infectious diseases underlines the necessity of correction for the decrease in notifications. As it is important for communicable disease professionals to act validly in a timely manner. Not correcting for the decrease of notifications during the COVID-19 pandemic could lead to false positive exceedances of the alarm threshold, thereby consuming time and other resources of communicable disease control professionals. The findings also suggest that certain outbreaks could potentially go undetected, and consequently, no action would be taken, with all its associated consequences. Furthermore, as for pandemic preparedness, data-driven decision and policy-making is crucial. Consequently, a robust surveillance system is a necessity. This study shows that correction for a decrease in notifications affects outbreak detection for other infectious diseases, even during an ongoing pandemic. In conclusion, it is recommended to account for periods of underreporting or reduced notifications to ensure reliable outbreak detection. Future work should explore which correction method is the most appropriate for outbreak detection in the PHS setting.

### Limitations

One of the limitations of this study is the inability to determine the factors driving the decrease in notifications based on this analysis. We hypothesized that the NPIs had an effect on the notifications, as the interference of the SARS-CoV-2 variants, possible shift in demographic determinants, and difference in health care use and access to diagnostics during the COVID-19 pandemic. We did not have any data on vaccination status, health care usage, and number of diagnostic requests; therefore, we could not identify if the notifications were affected by one of these factors specifically. However, we did not see any difference in the distribution of demographic determinants among the notifications. Other studies suggest that the decrease is likely due to a combination of factors [7,10,11]. In addition, exploring the causes of this decrease was beyond the scope of this study.

Another limitation is that we had a simplified definition for the post-COVID-19 pandemic period. We defined September 2022 as the end of the COVID-19 pandemic in the Netherlands, as the COVID-19 Stringency Index was at its lowest. One could argue that the post-COVID-19 pandemic period started later on, which could lead to underestimating the effect in the post-COVID-19 pandemic period. An alternative approach is to evaluate whether the monthly value is significantly below the historical average, similar to the methodology used for outbreak correction. As it will correct for short-term effects, this could create an alarm threshold that is more sensitive, which can be preferred in infectious disease control. Furthermore, assessing a monthly or even weekly correction approach would be valuable during a future pandemic, as it allows continuous adjustments for the ongoing impact rather than defining it retrospectively. This option was not assessed in our study, as we believe it would require more data and a prolonged study period. However, we recommend including this in future validation studies to further develop the “pandemic-proof” methodology.

Finally, we did not perform an interrupted time-series analysis for determining the trend of the notifications, as seen in other studies. This analysis required more data points than were available for some infectious diseases. Nevertheless, the negative binomial regression proved to be a robust alternative, as it accounts for zero notifications. To assess sensitivity, we compared the IRR between the pre-COVID-19 pandemic period and the entire COVID-19 pandemic period (2020‐2022) and found no notable differences to the IRRs of the individual COVID-19 pandemic periods. Our main focus was to identify which infectious diseases were affected and the duration of these effects, which was adequately addressed using the negative binomial regression.

### Conclusions

The COVID-19 pandemic and the related pandemic measures are likely to have had a considerable effect on the notifications of other infectious diseases, affecting both surveillance and outbreak detection. The length of the effect also differed among the infectious diseases, necessitating pattern-specific corrections. Our study shows that correcting for the decrease in notifications has an effect on the alarm thresholds for outbreak detection. Further research is required to determine which correction method is most suitable and applicable for effective outbreak detection, to ensure communicable disease control professionals act in a timely and data-driven manner.

## Supplementary material

10.2196/73953 Multimedia Appendix 1 Overview of hypothesized effects of factors on the number of notifications of listed infections during the COVID-19 pandemic, the Netherlands.

10.2196/73953 Multimedia Appendix 2 Descriptives of 25 notifiable infectious diseases (74,990 notifications) by the COVID-19 periods (pre-COVID, 2020, 2021, 2022, and post-COVID), 2015-2023, the Netherlands.

10.2196/73953 Multimedia Appendix 3 Number of monthly notifications of Hepatitis A with the unadjusted threshold and 3 corrected threshold methods for outbreak detection, The Netherlands, January 2015-December 2023.

10.2196/73953 Multimedia Appendix 4 Number of monthly notifications of measles with the unadjusted threshold and 3 corrected threshold methods for outbreak detection, The Netherlands, January 2015-December 2023.

10.2196/73953 Multimedia Appendix 5 Number of monthly notifications of mumps with the unadjusted threshold and 3 corrected threshold methods for outbreak detection, The Netherlands, January 2015-December 2023.

10.2196/73953 Multimedia Appendix 6 Number of monthly notifications of paratyphoid fever with the unadjusted threshold and 3 corrected threshold methods for outbreak detection, The Netherlands, January 2015-December 2023.

10.2196/73953 Multimedia Appendix 7 Number of monthly notifications of Q-fever with the unadjusted threshold and 3 corrected threshold methods for outbreak detection, The Netherlands, January 2015-December 2023.

10.2196/73953 Multimedia Appendix 8 Number of monthly notifications of typhoid fever with the unadjusted threshold and 3 corrected threshold methods for outbreak detection, The Netherlands, January 2015-December 2023.

10.2196/73953 Multimedia Appendix 9 Number of monthly notifications of meningococcal infection with the unadjusted threshold and 3 corrected threshold methods for outbreak detection, The Netherlands, January 2015-December 2023.

10.2196/73953 Multimedia Appendix 10 Number of monthly notifications of shigellosis with the unadjusted threshold and 3 corrected threshold methods for outbreak detection, The Netherlands, January 2015-December 2023.

10.2196/73953 Multimedia Appendix 11 Number of exceedances of the outbreak detection threshold for the unadjusted alarm threshold and the 3 correction methods, March 2020-December 2023.

10.2196/73953 Multimedia Appendix 12 Unadjusted alarm threshold and the alarm thresholds of the 3 correction methods for pertussis, March 2021-December 2023, the Netherlands

10.2196/73953 Multimedia Appendix 13 Unadjusted alarm threshold and the alarm thresholds of the 3 correction methods for malaria, March 2021-December 2023, the Netherlands.

## Abbreviations

Hib: *Haemophilus influenzae* type b
IRR: incidence rate ratio
NPI: nonpharmaceutical intervention
PHS: public health service
RIVM: National Institute for Public Health and the Environment
