# Supplementary figures and images for: A “Pandemic-Proof” Methodology for Outbreak Detection Adapted From COVID-19’s Impact on Notifications of Infectious Diseases in the Netherlands: Surveillance Study
Source: JMIR Public Health Surveill. 2025 Aug 26;11:e73953. doi: 10.2196/73953 (PMC12380364; doi:10.2196/73953)

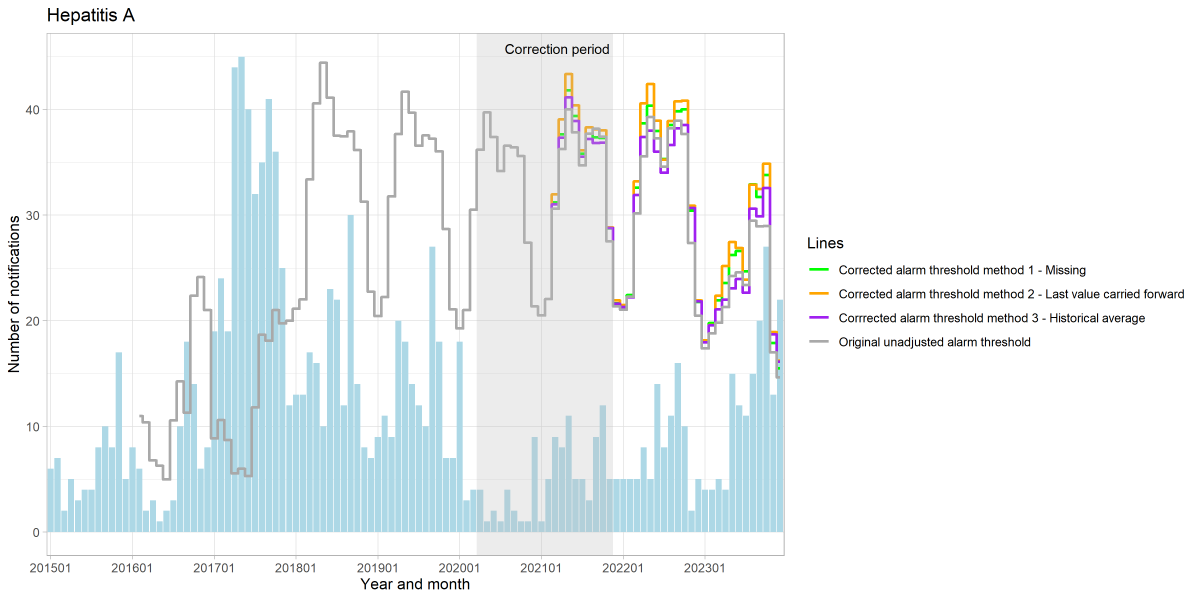

Supplement: Multimedia Appendix 3 [file publichealth-v11-e73953-s003.png]

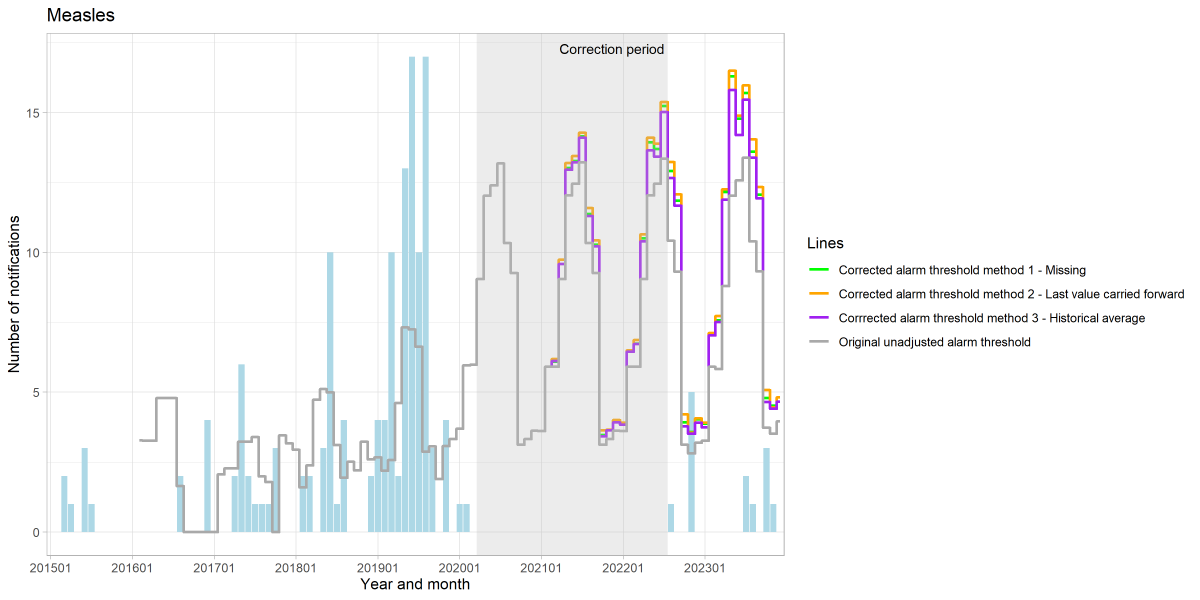

Supplement: Multimedia Appendix 4 [file publichealth-v11-e73953-s004.png]

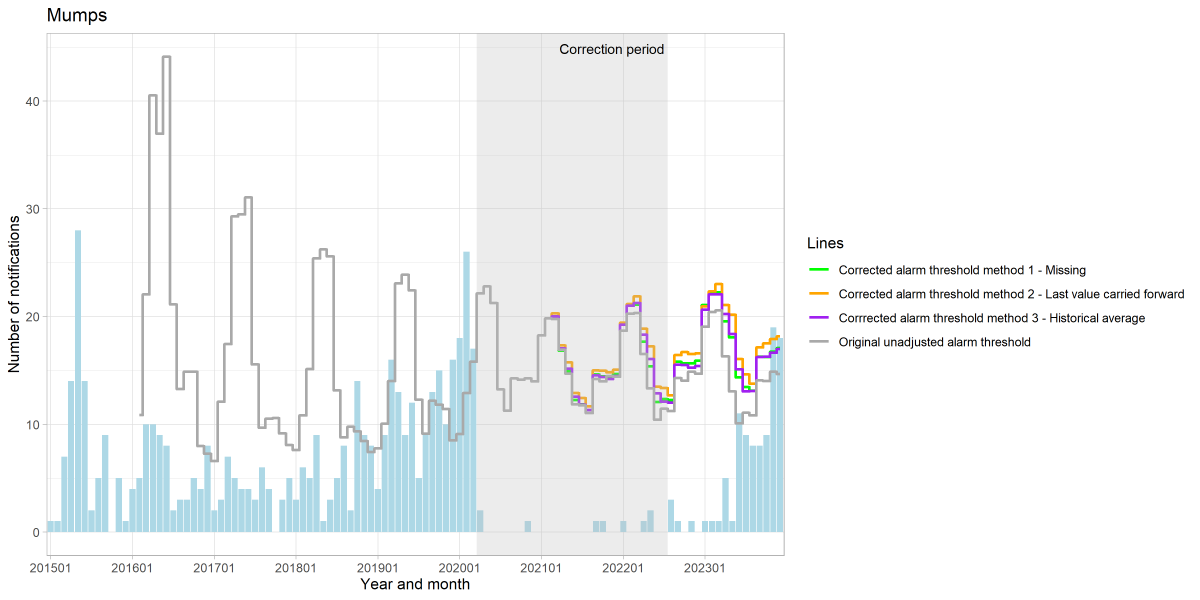

Supplement: Multimedia Appendix 5 [file publichealth-v11-e73953-s005.png]

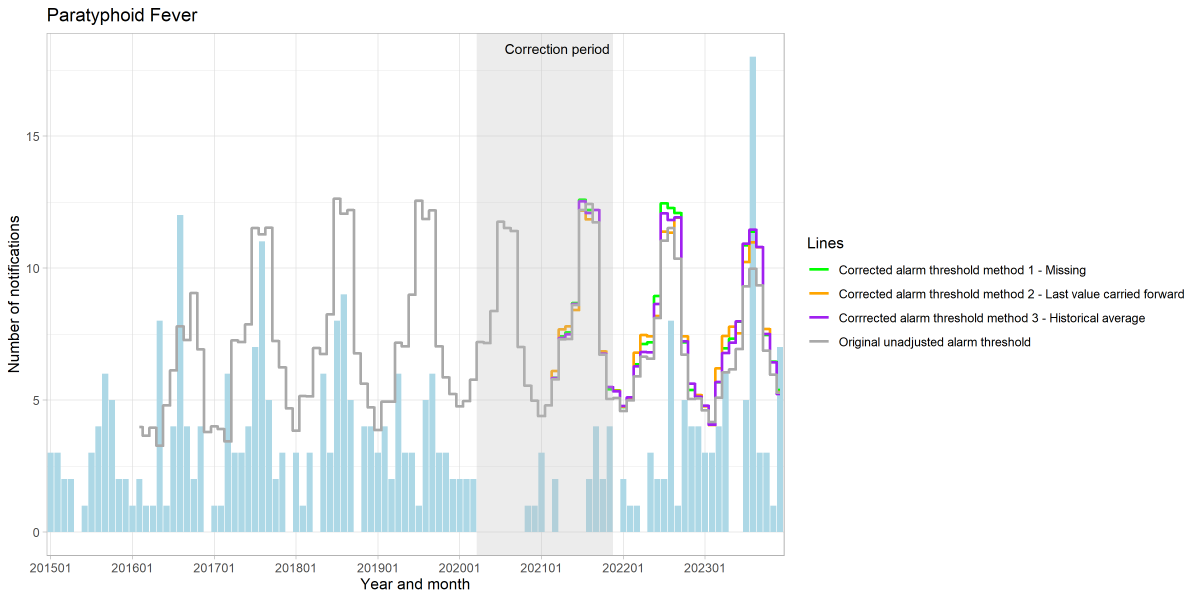

Supplement: Multimedia Appendix 6 [file publichealth-v11-e73953-s006.png]

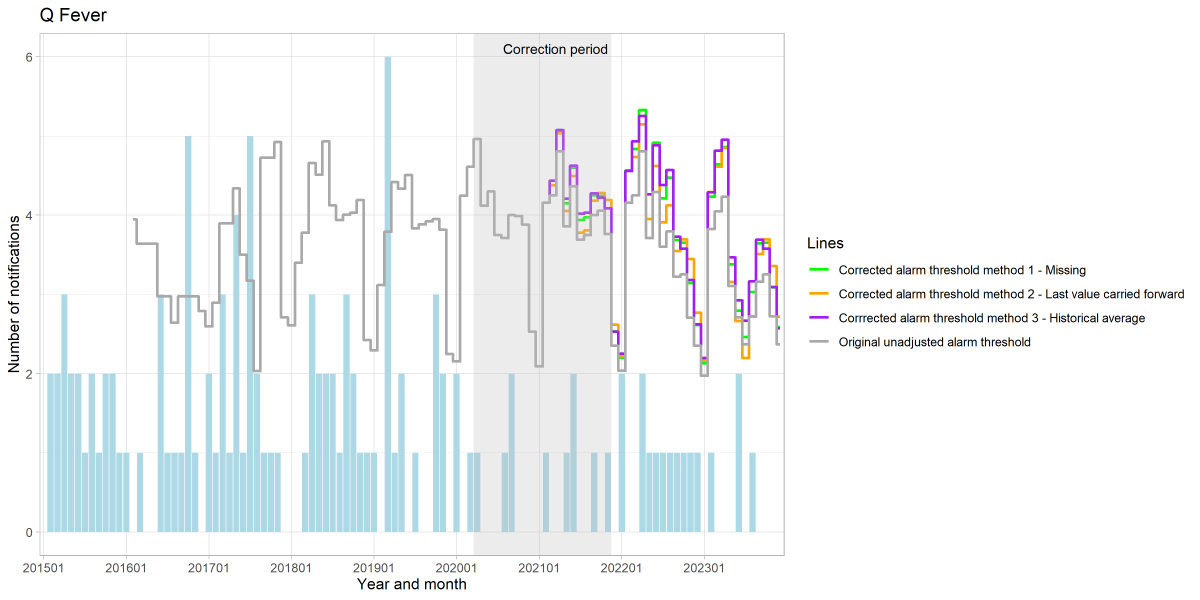

Supplement: Multimedia Appendix 7 [file publichealth-v11-e73953-s007.png]

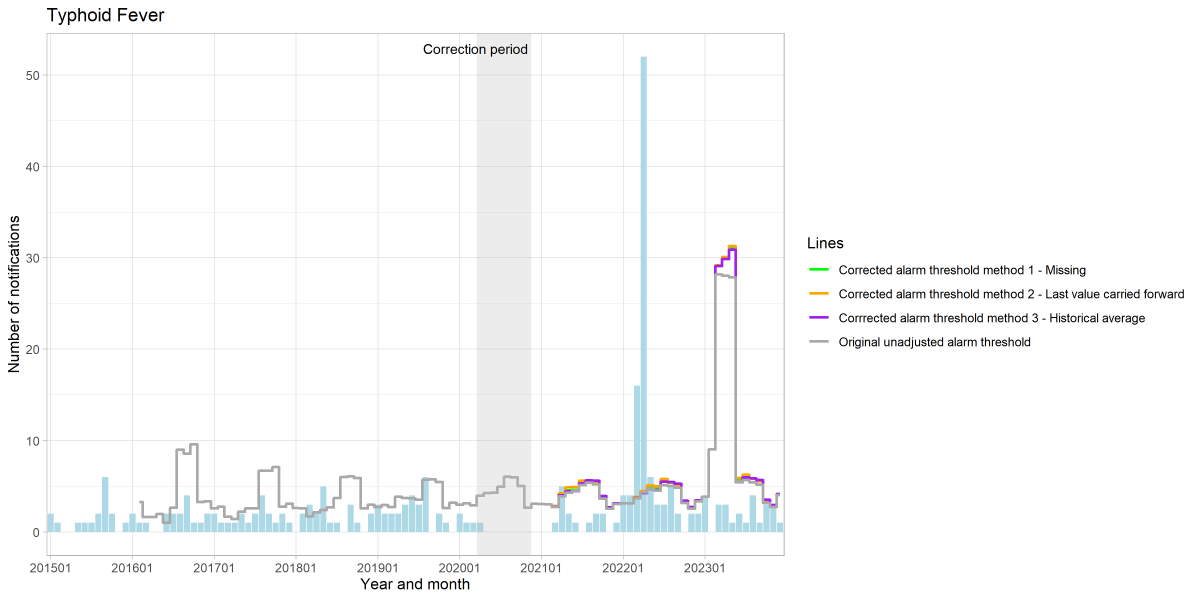

Supplement: Multimedia Appendix 8 [file publichealth-v11-e73953-s008.png]

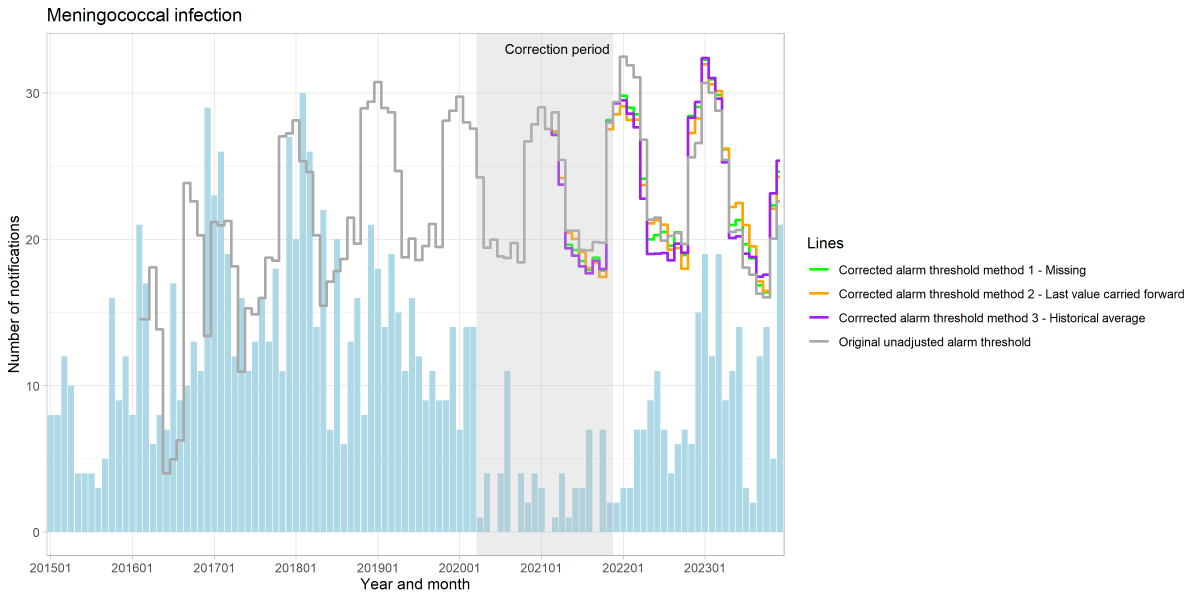

Supplement: Multimedia Appendix 9 [file publichealth-v11-e73953-s009.png]

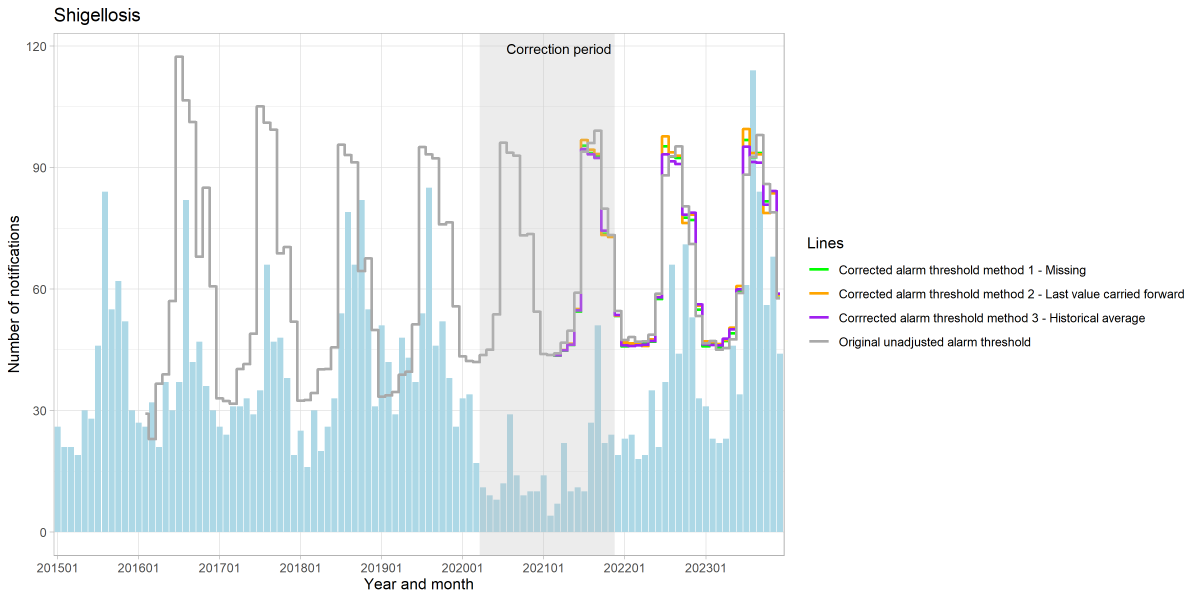

Supplement: Multimedia Appendix 10 [file publichealth-v11-e73953-s010.png]
