# Supplementary material for: A “Pandemic-Proof” Methodology for Outbreak Detection Adapted From COVID-19’s Impact on Notifications of Infectious Diseases in the Netherlands: Surveillance Study
Source: JMIR Public Health Surveill. 2025 Aug 26;11:e73953. doi: 10.2196/73953 (PMC12380364; doi:10.2196/73953)
Supplement: Multimedia Appendix 11 [file publichealth-v11-e73953-s011.pdf]

	Number of exceedances of the threshold since start COVID-19 pandemic			
**Infectious disease**	**Reference: uncorrected threshold**	**Option 1: Missing**	**Option 2: Last value carried forward**	**Option 3: Historical moving average**
**Affected during COVID 2020**				
Malaria	3	4	4	3
Typhoid Fever	8	8	8	8
**Affected during COVID 2020 & 2021**				
Hepatitis A	1	1	1	1
Meningococcal infection	0	0	0	0
Shigellosis	1	1	1	1
Paratyphoid Fever	2	2	2	2
Q-fever	0	0	0	0
**Affected during COVID 2020-2022**				
Measles	1	1	1	1
Mumps	3	2	1	2
Pertussis	1	1	1	1
